# Number of Premature Ventricular Complexes Predicts Long-Term Outcomes in Patients with Persistent Atrial Fibrillation

**DOI:** 10.3390/biomedicines12061149

**Published:** 2024-05-23

**Authors:** Kun-Chi Yen, Yi-Hsin Chan, Chun-Li Wang

**Affiliations:** 1 Division of Cardiology, Department of Internal Medicine, Chang Gung Memorial Hospital, Linkou Branch, Taoyuan 33305, Taiwan; kunchiyen@gmail.com (K.-C.Y.); wang3015001@gmail.com (C.-L.W.); 2 College of Medicine, Chang Gung University, Taoyuan 33302, Taiwan; 3 School of Traditional Chinese Medicine, College of Medicine, Chang Gung University, Taoyuan 33302, Taiwan; 4 Microscopy Core Laboratory, Chang Gung Memorial Hospital, Linkou, Taoyuan 33305, Taiwan

**Keywords:** atrial fibrillation, cardiovascular mortality, premature ventricular complex, ventricular arrhythmia, 24 h ECG

## Abstract

Background: Premature ventricular complexes (PVCs) are common electrocardiographic abnormalities and may be a prognosticator in predicting mortality in patients with structurally normal hearts or chronic heart diseases. Whether PVC burden was associated with mortality in patients with chronic atrial fibrillation (AF) remained unknown. We investigated the prognostic value of PVC burden in patients with persistent AF. Methods: A retrospective analysis of 24 h Holter recordings of 1767 patients with persistent AF was conducted. Clinical characteristics, 24 h average heart rate (HR), and PVC measures, including 24 h PVC burden and the presence of consecutive PVCs (including any PVC couplet, triplet, or non-sustained ventricular tachycardia) were examined for the prediction of all-cause and cardiovascular mortality using the Cox proportional hazards model. Results: After a median follow-up time of 30 months, 286 (16%) patients died and 1481 (84%) patients survived. Multivariate analysis revealed that age, heart failure, stroke, angiotensin-converting enzyme inhibitor/angiotensin receptor blocker, beta-blocker, digoxin, oral anticoagulant use, and estimated glomerular filtration rate were significant baseline predictors of all-cause mortality and cardiovascular mortality. Twenty-four-hour PVC burden and the presence of consecutive PVCs were significantly associated with all-cause and cardiovascular mortality after adjusting for significant clinical factors. When compared to the first quartile of PVC burden (<0.003%/day), the highest quartile (>0.3%/day) was significantly associated with an increased risk of all-cause mortality (hazard ratio, 2.46; 95% CI, 1.77–3.42) and cardiovascular mortality (hazard ratio: 2.67; 95% CI, 1.76–4.06). Conclusions: Twenty-four-hour PVC burden is independently associated with all-cause and cardiovascular mortality in patients with persistent AF.

## 1. Introduction

Premature ventricular complex (PVC) refers to the premature activation of the ventricular myocardium, a phenomenon frequently found in patients with both healthy and diseased hearts [1]. The prevalence of PVCs in a 10 s 12-lead electrocardiogram (ECG) is estimated to be between 1% and 4% in patients without heart disease [2,3]. However, the frequency of PVCs is notably increased when using ambulatory electrocardiogram (ECG) recordings, with 40% to 75% of participants experiencing PVCs during 24 to 48 h ambulatory Holter monitoring [4]. This difference can be attributed to a significant variability in PVC frequency with time. Prior research has shown that an elevated PVC burden is linked to occurrences of sudden cardiac death, cardiovascular (CV) events, cardiomyopathy, and ischemic stroke [5,6]. Additionally, the presence of multiform PVC patterns may be associated with cardiovascular adverse events in both healthy individuals and those with heart disease [7,8]. Nevertheless, the influence of the frequency of premature ventricular complex (PVC burden) on cardiovascular risks was identified in both structurally normal and diseased hearts with sinus rhythm [1,6,9,10].

Atrial fibrillation (AF) is the prevailing cardiac arrhythmia worldwide, affecting around 2% to 3% of the population. Moreover, the incidence of AF is considerably greater in those with heart conditions such as myocardial infarction or heart failure [11]. The predictive significance of PVCs in AF patients is still uncertain. Although the adverse effects of PVCs on cardiovascular function in patients with sinus rhythm are extensively researched, the rhythm irregularity of AF makes it difficult to accurately measure and understand the influence of PVCs on this condition. A recent study has demonstrated a correlation between AF and increased mortality in patients with existing ventricular tachyarrhythmias, specifically ventricular tachycardia (VT) and ventricular fibrillation (VF) [12]. Nevertheless, the precise correlation between PVCs burden and AF has yet to be clearly established in the current field of research. This study aims to bridge this gap by evaluating the influence of PVC burden in patients with persistent AF, based on 24 h electrocardiography (ECG) monitoring, on the cardiovascular and all-cause mortality during a long-term clinical follow-up.

## 2. Materials and Methods 

### 2.1. Study Population

This retrospective, single-center observational study was conducted following the Declaration of Helsinki and received approval from Chang Gung Memorial Hospital’s institutional review board. From 1 January 2010 to 31 December 2015, a total of 3261 patients with AF (paroxysmal or persistent) episodes were identified from the 24 h Holter ECG (Zymed system, Philips Healthcare) reporting during the outpatient or inpatient visit. AF episodes were defined as the irregular ventricular response in the absence of p waves or with fibrillatory waves. Paroxysmal AF was defined as any AF episode lasting more than 30 s and the remaining sinus rhythm recorded within the 24 h Holter ECGs. Persistent AF was defined as having over 99% AF events during 24 h Holter monitoring. We excluded those patients with a history of cardiac implantable electronic device implantation or evident artificial pacing spikes in the Holter data (n = 482). We excluded from the analysis participants who were classified as paroxysmal AF based on the Holter ECG recording (n = 1012). Finally, a cohort of 1767 patients with persistent AF was established for the present analysis. Over a median follow-up duration of 30 months (interquartile range, 14 to 47 months), 286 (16%) patients reached the designated study endpoint classified as all-cause death and 1481 (84%) patients remained as survived. The Zymed system by Philips Healthcare was employed for centralized data processing, with an experienced technician implementing manual modifications and a qualified electrophysiologist accomplishing a final review. The electrophysiologist was blind to the patient’s outcomes during the review process. The primary endpoint was cardiovascular and all-cause mortality. 

### 2.2. Statistical Analysis

Continuous variables were expressed as the mean ± standard deviation or the median with the range. Proportions were compared using *χ*^2^ tests. Correlations between continuous variables were assessed using linear regression. The univariate and multivariate Cox proportional hazard regression models were used to estimate the predictive variables for the outcome. Variables with a univariate statistical significance below 0.1 were selected to be included in the multivariate model. Statistical significance was defined as a *p*-value below 0.05 across all tests. The statistical analysis was performed using SPSS version 19.0 (SPSS Inc, Chicago, IL, USA).

## 3. Results

### 3.1. Baseline Characteristics and Outcomes

The baseline patient characteristics according to outcomes are summarized in Table 1. The mean QRS and QTc durations of the standard resting 12-lead ECG for the overall patients were 98.1 ± 24.1 and 454.1 ± 44.7 milliseconds (ms), respectively. For the overall 1767 patients with persistent AF, there were 455 (25.7%), 60 (3.4%), and 36 (2.0%) patients receiving an anti-arrhythmic drug, catheter ablation, and electrical cardioversion after the Holter ECG monitoring. There were 684 patients (38.7%) receiving digoxin treatment. For those treated with digoxin therapy, there were 258 patients with a diagnosis of congestive heart failure (90 patients with left ventricular ejection fraction of >40%). Two hundred and eighty-five patients treated with digoxin received digoxin level monitoring (mean level was 0.59 ± 0.44 ng/mL (effective level: 0.8–2.0 ng/mL)). For the overall study population, there were 1148 patients (65.0%) receiving oral anticoagulant treatment (547 and 601 patients receiving warfarin and direct oral anticoagulants (DOACs), respectively). Two hundred and eighty-six (16.2%) patients reached the study endpoint with all-cause mortality over a median follow-up period of 30 (interquartile range, 14–47) months. One hundred and seventy-six patients died of cardiovascular diseases (82 of pumping failure, 3 of acute pulmonary embolism, 38 of ventricular arrhythmias or pulseless electrical activity, 37 of acute cerebral vascular disease, and 16 of acute coronary syndrome). Patients reaching the endpoint were older and had a higher prevalence of comorbidities, higher CHA_2_DS_2_-VASc scores [13], and lower glomerular filtration rates. Patients who survived had a greater incidence of beta-blocker and anticoagulant use and a lower prevalence of digoxin usage. Regarding the 24 h Holter measures, patients who encountered the endpoints had a greater overall burden of PVCs, higher numbers of isolated PVCs, more episodes of PVC couplets, and instances of non-sustained ventricular tachycardia (defined as four or more consecutive beats originating below the atrioventricular node with an RR interval of less than 600 milliseconds, or more than 100 beats per minute, and lasting less than 30 s) during the 24 h Holter recording compared to those who did not reach the endpoint. Among the overall study cohort, there were 103 patients with a diagnosis of ventricular tachycardia runs (four or more consecutive PVC beats with an RR interval of less than 600 milliseconds) obtained from the 24 h Holter ECG recording. The median number of ventricular tachycardia runs for the 103 patients were five [interquartile range, 4–6] beats/24 h. There were 6 and 17 patients receiving implantable cardioverter-defibrillator implantation and catheter ablation after the diagnosis of ventricular tachycardia.

Univariate analysis utilizing a Cox proportional hazard model identified several characteristics correlated to outcomes, including age, heart failure, stroke, and a decreased glomerular filtration rate (Table 2). Using oral anticoagulants, beta-blockers, and ACEI/ARB medications was also associated with improved outcomes.

### 3.2. PVCs and Outcomes

In terms of the PVC measurements, the overall 24 h PVC burden and the occurrence of consecutive PVC episodes (including any PVC couplet, triplet, or ventricular tachycardia episode) were found to correspond to both all-cause and cardiovascular mortality, regardless of adjusting for baseline characteristics (as shown in Table 3).

**Figure 1:**
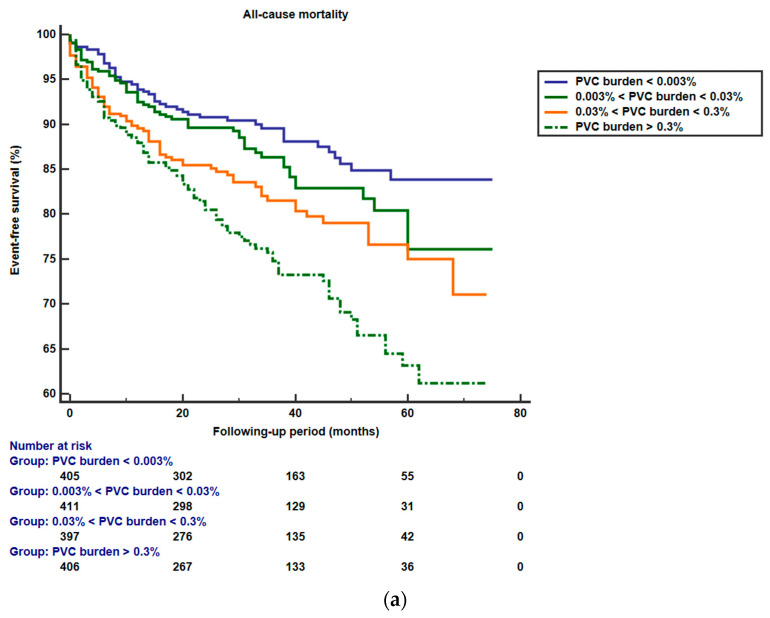

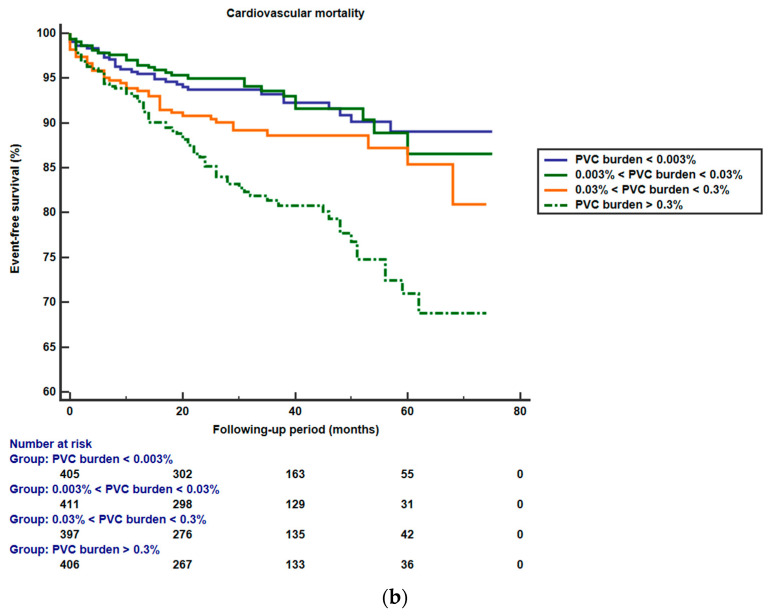
Kaplan–Meier curves of (**a**) all-cause and (**b**) cardiovascular mortality according to stratification of PVC burden.

Figure 1 displays the Kaplan–Meier curves representing the incidence of all-cause and cardiovascular mortality through PVC burden within 24 h. There was a noticeable increase in the risk of both all-cause and cardiovascular mortality in patients with persistent AF as the PVC burden rose from quartile 1 to 4. Compared to the lowest quartile of 24 h PVC burden (<0.002%/day), the highest quartile (>0.3%/day) demonstrated a significant association with an increased risk of all-cause mortality (hazard ratio, 2.43; 95% confidence interval, 1.73–3.40) and cardiovascular mortality (hazard ratio: 2.62; 95% confidence interval, 1.73–3.95).

**Figure 2:**
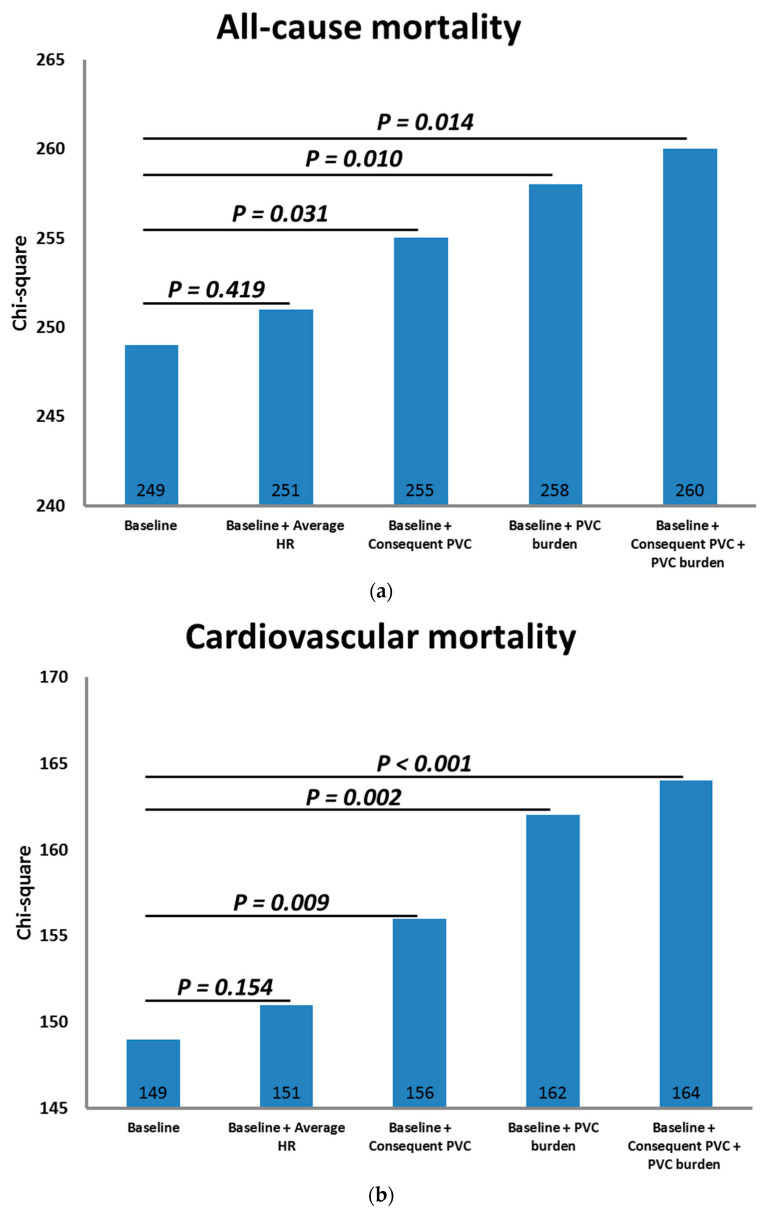
Chi-square test of prognostic value of 24 h average heart rate, PVC burden, and consecutive PVC episodes in predicting (**a**) all-cause and (**b**) cardiovascular mortality.

The initial model comprising the aforementioned significant variables could accurately predict all causes and cardiovascular mortality. The chi-square values for all-cause and cardiovascular mortality were 249 and 149, respectively, with a *p*-value less than 0.001. Figure 2 presents prognostic information obtained by incorporating the 24 h average heart rate, the 24 h burden of PVCs, and the presence of consecutive PVCs. Incorporating a 24 h burden of PVCs or the occurrence of consecutive PVCs significantly improved the ability to predict mortality compared to a model that only included baseline characteristics (both *p*-values are less than 0.05). Nevertheless, integrating the 24 h burden of PVCs and the presence of consecutive PVCs did not significantly improve the prediction performance. In contrast, the 24 h average heart rate did not yield any supplementary predictive information on overall and cardiovascular mortality compared to the initial model.

Table 4 presents the associations between PVC measurements and multiple variables in all study patients. The correlation between the 24 h burden of PVCs and baseline characteristics, such as age, estimated glomerular filtration rate (eGFR), left ventricular ejection fraction (LVEF), and CHA2DS2-VASc score, is weak (all correlation coefficient is less than 0.2). 

## 4. Discussions

Our investigation found that individuals with a higher 24 h PVC burden (>0.3%/day) had a significantly higher risk of all-cause and cardiovascular mortality compared to those with a lower 24 h PVC burden (<0.002%/day). The hazard ratios for each were 2.43 and 2.62, respectively. In addition, the examination of baseline data showed that the occurrence of consecutive PVC events (including any PVC couplet, triplet, or ventricular tachycardia episode) increased the risk of both all-cause and cardiovascular mortality. The adjusted hazard ratios were 1.30 and 1.50, respectively. The results of our study provide significant insights into the 24 h PVC burden in patients with AF, an issue that prior studies have not thoroughly investigated. 

Several studies have found an association between high PVC burden and LV dysfunction, as well as an increased risk of systolic HF (hazard ratio [HR]: 1.48 to 1.8) and mortality (HR: 1.31) [6,14,15,16,17,18] even after adjusting for age and other ECG abnormalities [19]. Researchers also found that in patients without substantial cardiac abnormalities, there is a range of PVC burden that is linked to higher mortality and hospitalization rates for cardiovascular issues. This range starts at a relatively low threshold of 12 PVCs per day [20]. Other research has encompassed a variety of PVC burdens ranging from 0.123% to 17.7%, or from 1000 to 10,000 per day and more than 10,000 per day, respectively. These discoveries demonstrate the intricate correlation between PVC burden and cardiovascular outcomes. Our study demonstrates that a higher PVC burden in patients with atrial fibrillation significantly correlates with elevated overall and cardiac mortality. We are expanding our understanding of the extensive impacts of PVC burden, affecting both patients with sinus rhythm and those with atrial fibrillation. This highlights the importance of implementing proactive measures to manage AF patients who experience frequent PVCs. This could include frequent surveillance, medication treatment [21], and, potentially, the application of ablation therapy, which has demonstrated efficacy in enhancing systolic function in cases with a high PVC burden (>10%/day in general) [16,19,22,23,24]. 

Recent animal studies demonstrated that the deterioration in left ventricular systolic function is considered to be associated with left ventricular dyssynchrony and eccentric hypertrophy triggered by PVCs [25,26]. A swine model of PVC-induced cardiomyopathy demonstrated persistent myocardial fibrosis and left ventricular dyssynchrony even after PVCs had been eliminated [27]. PVC-induced left ventricular fibrosis and dyssynchrony have been shown to contribute to a worsening cardiovascular outcome [28,29]. Researchers also investigated the influence of PVC coupling intervals on the occurrence of PVC-induced cardiomyopathy (PVC-CM) and cardiovascular mortality. Some studies show that interpolated PVCs [30] or short coupling intervals (RR′/RR ≤ 0.6) [31] may be a predictor of PVC-CM and restoration of left ventricular ejection fraction after catheter ablation [23]. At the same time, other studies have found that variability (dispersion) in PVC coupling intervals is correlated with both a higher risk of PVC-CM and a higher risk of cardiovascular mortality [32,33,34]. Animal studies have provided a plausible explanation for this phenomenon, indicating that PVCs at various coupling intervals disturb the function of cardiac neurons and the local reflex of the heart [35]. Our study focused specifically on patients diagnosed with atrial fibrillation. It is essential to consider the inherent variability in RR intervals common in these patients. This variation could contribute to a greater dispersion of PVC coupling intervals in patients with atrial fibrillation, providing an additional explanation for the observed increase in cardiovascular mortality in our study group. Additional research is required to specifically analyze the relationship between PVC coupling intervals and the occurrence of PVC-induced heart failure or cardiovascular mortality in AF patients.

Nevertheless, recent studies have indicated patients with PVCs who underwent thorough tests to eliminate the possibility of underlying cardiac disease did not see a deterioration in their prognostic outcomes during a follow-up period of 5.2 years [36]. This implies that patients who suffered from PVCs alone are not indicative of a higher risk of cardiovascular mortality. A recent study has found that there is a correlation between the presence of PVCs and the development of new-onset AF, especially in younger patients [37]. Consequently, performing a thorough assessment to exclude structured or underlying cardiac diseases, even atrial fibrillation, in patients experiencing PVCs, particularly those with a significant burden, is beneficial in distinguishing the group of patients at greater risk of poor clinical outcomes. Our study found that individuals with AF who had a more significant burden of PVCs are at a considerably increased risk of both all-cause and cardiovascular mortality. AF may contribute to obvious and subclinical changes in ventricular structure and function. Various potential pathological mechanisms have been proposed, such as the progression of ventricular rate, dysfunction of microvascular or endothelial cells, systemic inflammation causing reduced myocardial perfusion, abnormal calcium handling, and irregular atrioventricular conduction [38,39]. These factors are susceptible to reduced blood flow in both atria and ventricles, causing ischemia and the development of fibrosis and cardiac remodeling [39]. In addition, AF may trigger long–short sequences, and premature ventricular complexes can also lead to the presence of arrhythmogenic substrates that increase the risk of developing ventricular tachyarrhythmias such as VT or VF during AF in patients [40,41]. Our research indicates that consecutive PVCs can serve as a prognostic indicator for both overall and cardiovascular mortality in patients with AF. This condition is also commonly observed in patients with sinus rhythm. This discovery raises a crucial question for future investigation about whether the predictive feature of consecutive PVCs applies only to patients with persistent AF, or if it also includes individuals with sinus rhythm. Addressing this inquiry will expand our comprehension of the roles of PVCs in different cardiac arrhythmias and offer valuable perspectives on potential therapeutic strategies. Moreover, further research is required to explore the correlations between PVC burden and different cardiovascular events in patients with persistent AF, potentially resulting in the formulation of customized therapeutic approaches for these specific patient groups.

In summary, our research offers novel perspectives on the predictive significance of PVCs in patients with persistent AF, implicating that close monitoring or even early intervention strategies may be advantageous for decreasing the possibility of unfavorable cardiovascular or all-cause mortality.

## 5. Study Limitations

This retrospective analysis is subject to several limitations. The present study employs a retrospective and observational approach, which omits crucial details concerning variables like alcohol or tobacco consumption, physical activity levels, and family medical history. The dataset failed to consider confounding factors such as blood hemoglobin levels or physical activity that may impact heart rate. The potential impact of medication adjustments, such as anticoagulants, anti-arrhythmic agents, or rate control managements, on heart rate and outcomes was not accounted for during the follow-up period. Therefore, it is necessary to proceed with precaution while interpreting our data. This is a retrospective and observational study using real-world data from the large hospital-based claim database in Taiwan. The study population in the present study mainly focused on AF patients with multiple comorbidities due to the nature of the hospital-based claim database. Whether our results can be extrapolated to other AF populations with a different etiology or mechanism (e.g., parasympathetic enhancement or exercise-induced AF) remains unclear. The present guidelines recommend the use of digoxin for the management of rate control in patients with AF, particularly those with concomitant heart failure. Use of digoxin and the digoxin concentration were independently associated with mortality in patients with AF, regardless of heart failure [42,43]. However, the intention of digoxin treatment (for heart failure management or AF rate control) was unclear in the present study due to the nature of the retrospective and observational study design. Further investigation is required to validate the influence of the 24 h PVC burden on mortality in individuals with persistent AF. There were 103 patients with a diagnosis of VT runs (four or more consecutive PVC beats) reported from the 24 h Holter ECG recording. However, those patients with VTs had a quite small VT burden (median number of 5 [interquartile range, 4–6] consecutive runs/24 h). There were only 6 and 17 patients receiving implantable cardioverter-defibrillator implantation and catheter ablation after the diagnosis of VT obtained from Holter recording. Because most patients did not receive further Holter follow-up or receive further intervention (e.g., device implantation, catheter ablation, or coronary intervention) regarding the VT burden management, we cannot perform further analysis evaluating the risk reduction of incidents related to the interventions that potentially lower the burden of ventricular arrhythmia. Furthermore, the differentiation between ventricular ectopy and aberrant ventricular conduction during AF may be difficult due to the limited lead number of 24 h Holter recording in the present study.

## 6. Conclusions

The results of our study demonstrate that the 24 h burden of premature ventricular contractions is a substantial and independent predictor of both all-cause and cardiovascular mortality in patients with persistent atrial fibrillation. Regular assessment of PVC burden with 24 h ECG monitoring is a feasible approach to identifying patients with a heightened risk.

## Figures and Tables

**Table 1:** Baseline characteristics of all patients.

	Survivors (n = 1481)	Non-Survivors (n = 286)	*p*-Value
**Clinical variables**			
Age, years	69.0 ± 12.9	77.0 ± 11.0	<0.001
Men, %	60.4	54.2	0.049
CHA_2_DS_2_-VASc	2.9 ± 1.8	4.2 ± 1.7	<0.001
Diabetes mellitus, %	25.9	35.3	0.001
Hypertension, %	57.5	65.7	0.010
Ischemic stroke, %	15.6	29.7	<0.001
Vascular disease, %	11.4	18.2	0.002
Heart failure, %	21.4	38.5	<0.001
eGFR (mL/min/1.73 m^2^)	75.2 ± 33.4	60.9 ± 36.6	<0.001
LVEF, %	59.4 ± 14.6	60.4 ± 15.2	0.342
Resting HR, bpm	81.0 ± 19.7	80.7 ± 21.9	0.769
Systolic blood pressure, mmHg	129.1 ± 22.0	129.2 ± 24.8	0.945
Diastolic blood pressure, mmHg	73.9 ± 14.9	76.6 ± 14.0	0.003
**Medications**			
Beta-blocker, %	68.4	61.7	0.029
ACEI or ARB, %	66.7	63.9	0.363
Non-dihydropyridine CCB, %	50.2	57.9	0.017
Digoxin, %	37.3	46.0	0.006
Anti-arrhythmic agent, %	26.2	23.5	0.349
Anticoagulant, %	67.7	51.0	<0.001
**24-h Holter**			
24-h average HR, bpm	82 ± 19	82 ± 21	0.983
Total PVC burden (%) *	0.03 (0.00–0.25)	0.09 (0.01–0.80)	<0.001
Total PVC numbers *	29 (2–268)	108 (6–949)	<0.001
Isolated PVC number *	27 (1–263)	100 (6–890)	<0.001
PVC couplet episodes	4.0 ± 11.0	6.8 ± 15.1	<0.001
PVC triplet episodes	0.6 ± 3.9	0.9 ± 5.6	0.33 ± 9
VT episodes	0.2 ± 1.3	3.0 ± 41.5	0.009

* Data are presented as median (25% to 75%). Other data are presented as mean ± standard deviation or numbers (percentage). Abbreviations and Acronyms: AF = atrial fibrillation; ACEI = angiotensin-converting enzyme inhibitor; ARB = angiotensin receptor blocker; CCB = calcium channel blocker; CHA_2_DS_2_-VASc = congestive heart failure, hypertension, age 75 years or older, diabetes mellitus, previous stroke/transient ischemic attack, vascular disease, age 65 to 74 years, female; ECG = electrocardiogram; eGFR = estimated glomerular filtration rate; HR = heart rate; LVEF = left ventricular ejection fraction; PVC = premature ventricular complex; VT = ventricular tachycardia.

**Table 2:** Baseline predictors for all-cause mortality.

	Univariate		Multivariate	
	Hazard Ratio (95% Confidence Interval)	*p*-Value	Hazard Ratio (95% Confidence Interval)	*p*-Value
Age, per 10 years	1.83 (1.63–2.05)	<0.001	1.65 (1.46–1.86)	<0.001
Hypertension	1.42 (1.11–1.81)	0.005		
Diabetes mellitus	1.50 (1.18–1.92)	0.001		
Heart failure	2.04 (1.61–2.58)	<0.001	1.98 (1.52–2.56)	<0.001
Stroke	1.92 (1.49–2.48)	< 0.001	1.83 (1.41–2.39)	<0.001
Vascular disease	1.54 (1.14–2.08)	0.005		
Glomerular filtration rate, per 10 mL/min/1.73 m^2^	0.87 (0.83–0.90)	<0.001	0.93 (0.89–0.97)	<0.001
Use of anticoagulants	0.46 (0.36–0.58)	<0.001	0.46 (0.36–0.58)	<0.001
Use of ACEI/ARB	0.80 (0.63–1.02)	0.075	0.61 (0.47–0.80)	<0.001
Use of beta-blockers	0.74 (0.58–0.94)	0.014	0.76 (0.59–0.97)	0.027
Use of non-dihydropyridine CCB	1.25 (0.99–1.58)	0.065		
Use of digoxin	1.30 (1.03–1.64)	0.026	1.45 (1.13–1.86)	0.004

The abbreviations as in Table 1.

**Table 3:** Cox regression analyses of the effect of PVC profiles on mortality.

	All-Cause Mortality				Cardiovascular Mortality			
	Crude Hazard Ratio (95% CI)	*p*-Value	Adjusted Hazard Ratio * (95% CI)	*p*-Value	Crude Hazard Ratio (95% CI)	*p*-Value	Adjusted Hazard Ratio † (95% CI)	*p*-Value
24 h average HR (per 10 BPM)	1.05 (0.97–1.13)	0.22	1.03 (0.97–1.09)	0.377	0.95 (0.87–1.03)	0.200	0.94 (0.88–1.03)	0.949
24 h PVC burden (%)	1.11 (1.04–1.17)	0.001	1.10 (1.03–1.17)	0.004	1.15 (1.08–1.22)	<0.001	1.14 (1.06–1.22)	<0.001
PVCs < 100/24-h	**Reference**	<0.001	**Reference**	<0.001	**Reference**	<0.001	**Reference**	<0.001
PVCs 100–1000/24-h	1.59 (1.21–2.10)		1.37 (1.04–1.81)		1.48 (1.03–2.14)		1.22 (0.84–1.77)	
PVCs > 1000/24-h	2.23 (1.67–2.97)		1.87 (1.39–2.51)		2.71 (1.90–3.85)		2.18 (1.52–3.11)	
PVC couplets episode	1.01 (1.01–1.02)	<0.001	1.01 (1.00–1.02)	0.013	1.02 (1.01–1.03)	<0.001	1.01 (1.00–1.02)	0.003
PVC triplets episode	1.00 (0.99–1.03)	0.417	1.00 (0.98–1.03)	0.917	1.01 (0.98–1.04)	0.484	1.00 (0.97–1.03)	0.918
VT episode	1.00 (1.00–1.01)	0.002	1.01 (1.00–1.01)	<0.001	1.01 (1.00–1.01)	<0.001	1.01 (1.00–1.01)	<0.001
Presence of consecutive PVCs (couplet, triplet or VT episode)	1.62 (1.28–2.04)	<0.001	1.30 (1.03–1.64)	0.031	1.89 (1.41–2.54)	<0.001	1.50 (1.11–2.03)	0.009

The abbreviations as in Table 1. * All-cause mortality adjusted for age, stroke, heart failure, eGFR, use of oral anticoagulants, beta-blocker, ACEI/ARB, and digoxin. † Cardiovascular mortality adjusted for age, stroke, heart failure, eGFR, use of oral anticoagulants, ACEI/ARB, and digoxin.

**Table 4:** Correlates of 24 h PVC number and PVC burden in study patients.

	24 h PVC Number		24 h PVC Burden	
	*r*	*p*-Value	*r*	*p*-Value
Age	0.120	0.06	0.128	<0.0001
CHA_2_DS_2_-VASc	0.140	<0.0001	0.144	<0.0001
eGFR	−0.076	0.0002	−0.076	0.0002
LVEF	−0.142	<0.0001	−0.099	<0.0001
24 h average HR	0.029	0.216	−0.039	0.107

The abbreviations as in Table 1.

## Data Availability

The datasets used in this study were only available in the Chang Gung Medical Data Center, Taiwan. The SPSS program (codes) involved for this study is available from the corresponding author on reasonable request.
